# Efficacy and Safety of Qili Qiangxin Capsule on Dilated Cardiomyopathy: A Systematic Review and Meta-Analysis of 35 Randomized Controlled Trials

**DOI:** 10.3389/fphar.2022.893602

**Published:** 2022-04-28

**Authors:** Jingjing Wei, Bin Li, Xinlu Wang, Xingyuan Li, Yucai Hu, Lijie Qiao, Cheng Zhou, Peng Yu, Tianqing Sang, Mingjun Zhu, Yongxia Wang

**Affiliations:** ^1^ First Affiliated Hospital of Henan University of CM, Zhengzhou, China; ^2^ Henan University of Chinese Medicine, Zhengzhou, China

**Keywords:** Qili Qiangxin capsule, dilated cardiomyopathy, randomized controlled trials, meta-analysis, grade

## Abstract

**Objective:** Qili Qiangxin Capsule (QQC), a Chinese patent medicine, is clinically effective in treating dilated cardiomyopathy (DCM). However, the meta-analysis of QCC combined with conventional western medicine (CWM) on DCM remains unexplored. This study aimed to systematically evaluate the efficacy and safety of QCC in the treatment of DCM.

**Methods:** Searched the studies of the combination of QQC and CWM in the treatment of DCM, from databases like PubMed, Cochrane Library, Web of Science, Wan Fang Databases, Chinese Biomedical Literature Database, China Science and Technology Journal Database, China National Knowledge Infrastructure, prior to 15 January 2022. Two reviewers respectively regulated research selection, data extraction, and risk of bias assessment. Review Manager Software 5.4 was used for meta-analysis. Furthermore, GRADE pro3.6.1 software was selected to grade the current evidence in our findings. This meta-analysis has been registered in PROSPERO (CRD42022297906).

**Results:** There were 35 studies pertaining to 3,334 patients included. The meta-analysis showed compared with CWM alone, the combination therapy had significant advantages in improving the clinical efficiency rate (RR = 1.24, 95% CI: 1.19 to 1.29, *p* < 0.00001), 6 min walking distance (6MWD) (MD = 41.93, 95%CI: 39.82 to 44.04, *p* < 0.00001), superior in ameliorating the left ventricular ejection fraction (LVEF) (MD = 5.73, 95%CI: 4.70 to 6.77, *p* < 0.00001), left ventricular end-diastolic dimension (LVEDD) (MD = −4.09, 95%CI: −4.91 to −3.27), *p* < 0.00001), left ventricular end-systolic diameter (LVESD) (MD = −4.73, 95%CI: −5.63 to −3.84), *p* < 0.00001) and BNP (MD = −101.09, 95%CI: -132.99 to −69.18), *p* < 0.00001), and also superior in reducing hypersensitive-C-Reactive Protein (hs-CRP) (MD = −3.78, 95%CI: −4.35 to −3.21), *p* < 0.00001), Interleukin- 6 (IL-6) (MD = −25.92, 95%CI: −31.35 to -20.50), *p* < 0.00001), tumor necrosis factor-α (TNF-α) (MD = -5.04, 95%CI: −6.13 to −3.95), *p* < 0.00001), high mobility group protein B1 (HMGB1) (MD = −4.34, 95%CI: −5.22 to −3.46), *p* < 0.00001), and adverse reactions (ARs) (RR = 0.70, 95%CI: 0.51–0.97), *p* = 0.03). The GRADE evidence quality rating presented with moderate or low quality of evidence for the available data.

**Conclusion:** Compared with the control group, QQC combined with CWM may be effective in treating DCM. However, the conclusion of this study must be interpreted carefully due to the inferior quality and ambiguity of bias in the included trials.

**Systematic Review Registration**: https://www.crd.york.ac.uk/prospero, identifier [CRD42022297906].

## Introduction

Dilated cardiomyopathy (DCM) is a heterogeneous cardiomyopathy characterized by enlarged ventricles and reduced myocardial systolic function (Reichart et al., 2019). DCM usually results from myocarditis, genetic and environmental insults, metabolic or endocrine disturbances, and neuromuscular disease (Japp et al., 2016). Notably, DCM is a major cause of heart failure, leading to sudden cardiac death in severe cases, and is the most common indication for heart transplantation worldwide. Of concern, DCM predominantly affects younger adults, with an incidence of 5–7 cases per 100,000 people per year (Weintraub et al., 2017). A report from China in 2014 showed that 767 cases of DCM had a mortality rate of 42.24% at 52 months of follow-up (Liu et al., 2014). More importantly, DCM not only significantly reduces the quality of life but also results in high admission and readmission accompanied by a heavy burden on society and families.

Current treatments for dilated cardiomyopathy, such as beta-blockers, angiotensin-converting enzyme inhibitors (ACE-I)/angiotensin II receptor antagonists (ARB), digoxin, spirolactone, and mechanotherapy, are aimed at reducing the rate of damage to the myocardium and not increasing its regenerating potential (Reichart et al., 2019). Consequently, it is highly required to seek out new therapeutic methods for this significant, unmet medical need. The growing utilization of complementary and alternative medicine, consisting of Chinese patent medicine in treating DCM, has recently attracted widespread attention.

Qili Qiangxin Capsule (QQC) is a drug clearly recommended in the “Chinese guidelines for the diagnosis and treatment of dilated cardiomyopathy,” which has been collected by the Pharmacopoeia of the People’s Republic of China (Chinese Society of Cardiology and Chinese myocarditis cardiomyopathy cooperative group, 2018). **QQC** comprises 11 crude herbs (Table 1). The results of pharmacological experiments showed that QQC can improve the heart function, inhibits cardiomyocyte apoptosis via activating peroxisome proliferation-activated receptor γ, reduce the high expression of MMP-2 and MMP-9 in cardiomyocytes, control the activation of the RAAS system in the paraventricular nucleus of the hypothalamus, reduce the activity of renal sympathetic nerves (Ma L. Y et al., 2016; Wu et al., 2021).

**TABLE 1:** The main components of Qili Qiangxin Capsule.

Formulation	Source	Species, family, genus	Quality control reported	Chemical analysis reported
Qili Qiangxin Capsule	Shijiazhuang Yiling Pharmaceutical Co., Ltd.	*Astragalus mongholicus* Bunge*.*	Pharmacopoeia of China, 2020 edition	Zhang et al. (2019)
		Family: Fabaceae Lindl.		
		Genus: *Astragalus* L.		
		*Panax ginseng* C.A.Mey.		
		Family: Araliaceae Juss.		
		Genus: *Panax* L.		
		*Aconitum carmichaelii* Debeaux		
		Family: Ranunculaceae Juss.		
		Genus: *Aconitum* L.		
		*Salvia miltiorrhiza* Bunge.		
		Family: Lamiaceae Martinov.		
		Genus: *Salvia* L.		
		*Descurainia sophia (L.) Webb ex Prantl.*		
		Family: Brassicaceae Burnett.		
		Genus: *Descurainia* Webb & Berthel.		
		*Alisma plantago-aquatica L.*		
		Family: Alismataceae Vent.		
		Genus: *Alisma* L.		
		*Polygonatum odoratum* (Mill.) Druce.		
		Family: Asparagaceae Juss.		
		Genus: *Polygonatum* Mill.		
		· *Neolitsea cassia* (L.) Kosterm.		
		Family: Lauraceae Juss.		
		Genus: *Neolitsea* Merr.		
		*Carthamus tinctorius* L.		
		Family: Asteraceae Bercht. and J.Presl.		
		Genus: *Carthamus* L.		
		*Periploca sepium Bunge.*		
		Family: *Apocynaceae Juss.*		
		Genus: *Periploca* Tourn. ex L.		
		*Citrus × aurantium* L		
		Family: Rutaceae Juss.		
		Genus: *Citrus* L.		

In recent years, a large number of randomized controlled trials (RCTs) have shown that QQC has a definite effect in the treatment of DCM. Nonetheless, the clinical evidence of QCC for DCM has not been established until now. This study aims to systematically evaluate the safety and effectiveness of QQC in the treatment of DCM.

## Methods

### Program and Registration

This meta-analysis was carried out strictly in accordance with the PRISMA guidelines (see Supplementary Table S1) and has been registered in PROSPERO (CRD42022297906).

### Study Inclusion and Exclusion Criteria

#### Types of Research

Only RCTs of QQC for patients with dilated cardiomyopathy were included and not restricted by language or publication status.

#### Types of Participants

All subjects of the included study meet the diagnostic criteria for DCM established by the American Heart Association or the Chinese Medical Association (Maron et al., 2006; Chinese Society of Cardiology and Chinese myocarditis cardiomyopathy cooperative group, 2018). Literature required complete safety and efficacy data with a balanced baseline and comparability. The patients’ age was over 18 years. Gender, nationality, and race were not limited.

#### Types of Interventions

Participants in the control group received conventional western medicine (CWM), including ACE-I/ARB, Beta-blockers, mineralocorticoid receptor antagonists, angiotensin receptor-neprilysin inhibitor, diuretics, digoxin, and other drugs recommended by the guidelines. The experimental group received QCC in addition to the CWM of the control group.

#### Types of Outcomes

The primary outcomes were the clinical efficiency rate, left ventricular ejection fraction (LVEF), left ventricular end-diastolic dimension (LVEDD), left ventricular end-systolic diameter (LVESD), and adverse reactions (ARs). The secondary outcomes were brain natriuretic peptide (BNP), 6 min walking distance (6MWD), hypersensitive-C-Reactive Protein (hs-CRP), Interleukin- 6 (IL-6), tumor necrosis factor-α (TNF-α), and high mobility group protein B1 (HMGB1).

#### Exclusion Criteria

The exclusion criteria included studies with incomplete data, repeated publications, or mismatched outcome indicators.

### Search Strategy

PubMed, Cochrane Library, Web of Science, Wan Fang Databases, Chinese Biomedical Literature Database (CBM), China Science and Technology Journal Database (VIP), China National Knowledge Infrastructure (CNKI) were searched and collected RCT studies of QQC in the treatment of DCM, from the date of establishment of each database to 15 January 2022. The search terms included “Qili Qiangxin capsule”, “Qili Qiangxin”, “qiliqiangxin”, “dilated cardiomyopathy”, “DCM”, “dilative cardiomyopathy.” The search strategies are presented in Supplementary Table S2. In addition, the reference lists of the identified original articles were manually checked for other eligible studies.

### Study Selection and Data Extraction

Two investigators independently screened according to the title and abstract of the records retrieved in all databases and further assessed the full text for potentially eligible studies. For studies with missing information, the investigators contacted the authors for confirmation. Two researchers separately extracted literature data, including the author, publication year, sample size, age, gender, intervention measures, treatment methods, and treatment time. In the event of a disagreement, the third party shall participate in the negotiation and settlement.

### Risk of Bias Assessment

Two independent authors assessed the methodological quality and risk of bias of the included RCTs using the Cochrane Collaboration Tool (Higgins et al., 2011). The evaluation includes seven types: random sequence generation, incomplete outcome data, allocation concealment, selective reporting, blinding of participants and personnel, blinding of outcome assessment, and other bias. Then, Review Manager 5.3 should be used to display the bias risk assessment chart drawn.

### Data Analysis

Revman 5.3 software was used for Meta-analysis. Binary variables were statistically analyzed by relative risk (RR), and continuous variables were statistically analyzed by mean difference (MD). Each effect size was evaluated with a 95% confidence interval (CI). Measure the statistical heterogeneity according to the value of *I* ^2^, and the evaluation results are shown in forest maps. The random-effects model is used for analysis if *I* ^2^＞50%. Otherwise, the fixed effects model is used for analysis. Sensitivity analysis or subgroup analysis was performed to explore potential sources of heterogeneity. Finally, funnel plots and Egger’s test examined the publication bias effect if more than ten studies were included in the meta-analysis.

### Evidence Quality Assessment

The Grading of Recommendations Assessment, Development, and Evaluation (GRADE) criteria were employed to assess the quality of the evidence (Balshem et al., 2011). The quality of evidence of the meta-analysis outcomes was categorized as either very low, low, moderate, or high. Initially, the RCT outcomes were ranked as high-quality evidence. The quality of each outcome independently assessed by two authors was de-graded due to the following factors: risk of bias, inconsistency, indirectness, imprecision, and publication bias. Data analysis and synthesis were performed using GRADE pro3.6.1 software.

## Results

### Literature Retrieval

**FIGURE 1:**
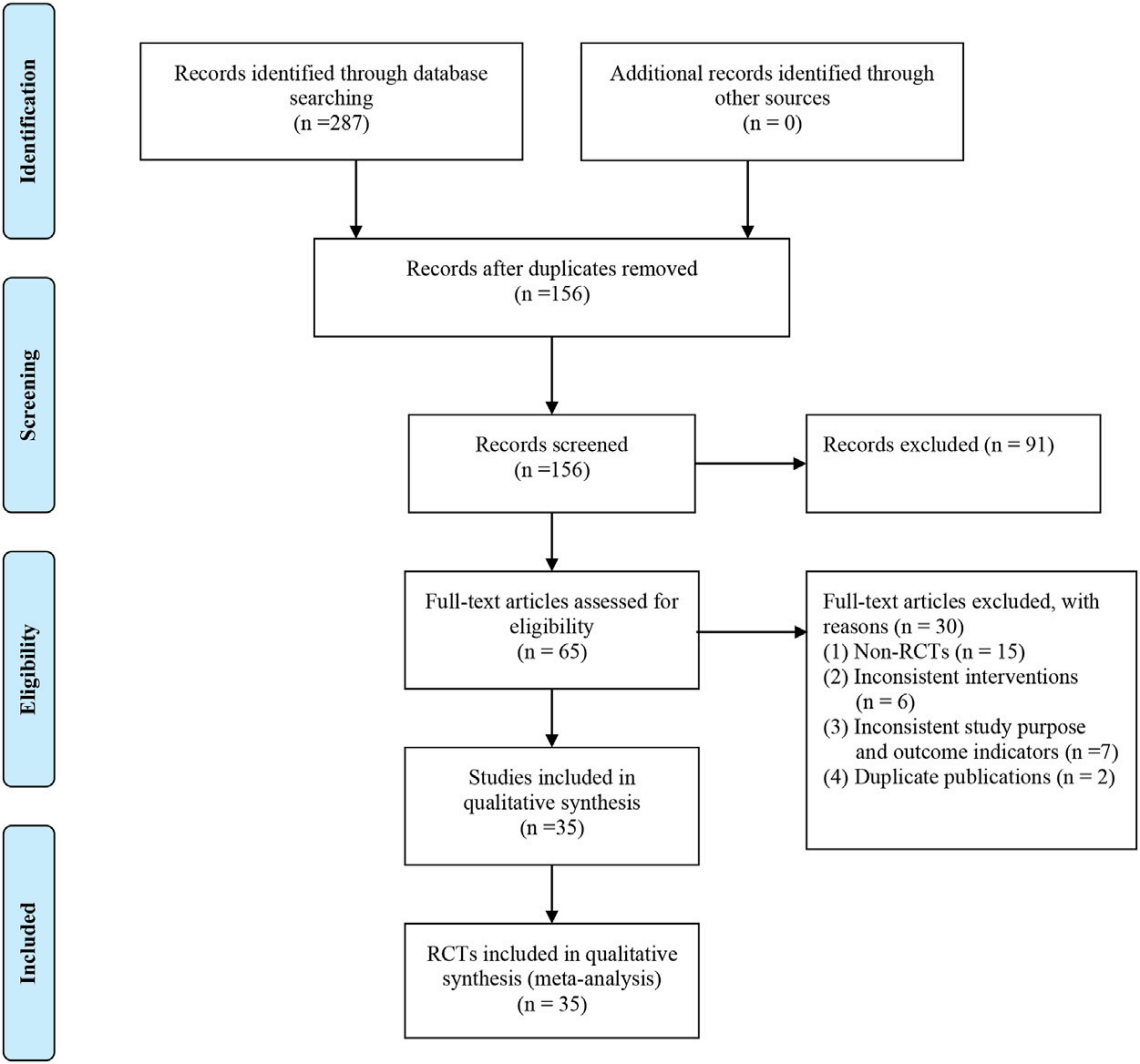
Flow diagram of study selection and identification.

Overall, 287 potentially relevant articles from seven electronic databases were retrieved after the literature search. After the elimination of duplicates, 156 articles were identified. After a detailed screening of the titles and abstracts, 91 articles including case reports, reviews, conference papers, and animal studies irrelevant to DCM were excluded. After reading the full text of the remaining 65 articles, 30 studies were further removed for at least one of the following reasons: non-RCTs (*n* = 15); inconsistent interventions (*n* = 24); inconsistent study purpose and outcome indicators (*n* = 7); and duplicate publications (*n* = 2). The final review included 35 studies (Liu et al., 2009; Zhu et al., 2010; Sun et al., 2012; Yuan, 2012; Yang et al., 2013; Zhang et al., 2013; Wu et al., 2014; Yi and Ou-yang, 2014; Ren, 2015; Zhou and Sun, 2015; Han, 2016; Leng, 2016; Ma X. Y et al., 2016; Jing et al., 2017; Fang, 2018; Hang, 2018; Wang, 2018; Wu et al., 2018; Yao, 2018; Zhao, 2018; Dai and Wang, 2019; Dong, 2019; Liu and He, 2019; Wang and Chang, 2019; Yang et al., 2019; Zheng et al., 2019; Li et al., 2020; Meng, 2020; Niu et al., 2020; Shi, 2020; Wang et al., 2020; Wang and Hu, 2020; Yao et al., 2020; Fan et al., 2021; Niu, 2021). The study selection process is shown in Figure 1.

### Basic Features of Literature Research

All of the trials were double-arm randomized clinical trials. The enrolled 35 articles were published between 2009 and 2021, in which all studies related to the comparison of QCC combined with CWM vs. CWM. The standard, type, and dose of conventional western medicine in the QCC group were the same as those in the control group. The composition of QQC was the same in all included studies in our study, and the dosage of QQC in the trial group was 1.2 g three times a day. A total of 3,334 patients were randomly divided into a QCC group and a control group, all from China, including 1850 men. The mean age of the participants ranged from 37.72 to 68.77 years. Treatment duration lasted 2 weeks to 12 months, and most of them lasted 4 weeks (8/35, 23%). The primary outcomes measure was reported. Among them, the clinical efficiency rate was reported in twenty-three studies (Liu et al., 2009; Sun et al., 2012; Zhang et al., 2013; Han, 2016; Ma X. Y et al., 2016; Jing et al., 2017; Fang, 2018; Hang, 2018; Wang, 2018; Yao, 2018; Dai and Wang, 2019; Dong, 2019; Liu and He, 2019; Wang and Chang, 2019; Zheng et al., 2019; Meng, 2020; Niu et al., 2020; Shi, 2020; Wang et al., 2020; Wang and Hu, 2020; Yao et al., 2020; Fan et al., 2021; Niu, 2021), LVEF in 28 (Liu et al., 2009; Zhu et al., 2010; Yuan, 2012; Yang et al., 2013; Zhang et al., 2013; Wu et al., 2014; Yi and Ou-yang, 2014; Ren, 2015; Zhou and Sun, 2015; Leng, 2016; Ma X. Y et al., 2016; Jing et al., 2017; Hang, 2018; Wu et al., 2018; Zhao, 2018; Dai and Wang, 2019; Dong, 2019; Liu and He, 2019; Wang and Chang, 2019; Yang et al., 2019; Zheng et al., 2019; Li et al., 2020; Meng, 2020; Niu et al., 2020; Shi, 2020; Wang et al., 2020; Wang and Hu, 2020; Yao et al., 2020; Fan et al., 2021), LVEDD in 20 (Liu et al., 2009; Zhu et al., 2010; Yuan, 2012; Yang et al., 2013; Zhang et al., 2013; Yi and Ou-yang, 2014; Zhou and Sun, 2015; Leng, 2016; Jing et al., 2017; Wu et al., 2018; Zhao, 2018; Dong, 2019; Liu and He, 2019; Yang et al., 2019; Zheng et al., 2019; Li et al., 2020; Meng, 2020; Wang et al., 2020; Wang and Hu, 2020; Fan et al., 2021), LVESD in 14 (Zhang et al., 2013; Yi and Ou-yang, 2014; Jing et al., 2017; Wu et al., 2018; Zhao, 2018; Dong, 2019; Liu and He, 2019; Yang et al., 2019; Zheng et al., 2019; Li et al., 2020; Meng, 2020; Wang et al., 2020; Wang and Hu, 2020; Fan et al., 2021), ARs in 11 (Liu et al., 2009; Yang et al., 2013; Leng, 2016; Fang, 2018,; Wang, 2018; Wu et al., 2018; Niu et al., 2020; Wang et al., 2020; Wang and Hu, 2020; Fan et al., 2021; Niu, 2021) (Table 2).

**TABLE 2:** Characteristics of the included trials.

Study ID	Sample size	Gender	Age (year)		NYHA classification	Duration	Interventions		QCC dosage	Outcomes
	(T/C)	(M/F)	T	C			T	C		
Niu (2021)	51/51	57/45	56.58 ± 5.76	56.60 ± 5.85	Ⅱ, Ⅲ, Ⅳ	3 months	QCC + CWM	CWM	1.2 g, t.i.d.	CER, ARs
Fan et al. (2021)	43/43	55/31	46.17 ± 7.15	60.12 ± 7.84	Ⅲ, Ⅳ	10 weeks	QCC + CWM	CWM	1.2 g, t.i.d.	CER, LVEF, LVEDD, LVESD, ARs
Yao et al. (2020)	48/48	53/43	50.43 ± 6.24	50.53 ± 6.12	Ⅱ, Ⅲ, Ⅳ	3 months	QCC + CWM	CWM	1.2 g, t.i.d.	CER, LVEF, hs-CRP, TNF-α
Wang et al. (2020)	45/41	57/29	54.4 ± 10.8	53.9 ± 11.3	Ⅱ, Ⅲ, Ⅳ	8 weeks	QCC + CWM	CWM	1.2 g, t.i.d.	CER, LVEF, LVEDD, LVESD, 6MWD, ARs
Meng (2020)	50/50	53/47	58.25 ± 3.67	58.25 ± 3.67	Ⅱ, Ⅲ, Ⅳ	2 weeks	QCC + CWM	CWM	1.2 g, t.i.d.	CER, LVEF, LVEDD, LVESD, BNP, 6MWD
Wang and Hu (2020)	40/40	50/30	68.63 ± 4.57	68.77 ± 7.16	Ⅱ, Ⅲ, Ⅳ	4 weeks	QCC + CWM	CWM	1.2 g, t.i.d.	CER, LVEF, LVEDD, LVESD, ARs
Shi (2020)	30/30	33/27	65.5 ± 3.4	65.4 ± 3.6	Ⅱ, Ⅲ	4 weeks	QCC + CWM	CWM	1.2g, t.i.d.	CER, LVEF
Niu et al. (2020)	68/67	83/52	47.05 ± 5.01	46.75 ± 5.01	Ⅱ, Ⅲ, Ⅳ	4 weeks	QCC + CWM	CWM	1.2 g, t.i.d.	CER, LVEF, 6MWD, ARs
Li et al. (2020)	40/40	53/27	54.58 ± 9.23	54.74 ± 9.36	Ⅱ, Ⅲ, Ⅳ	1 month	QCC + CWM	CWM	1.2 g, t.i.d.	LVEF, LVEDD, LVESD
Zheng et al. (2019)	52/52	57/47	61.00 ± 10.33	62.31 ± 12.07	Ⅱ, Ⅲ, Ⅳ	2 weeks	QCC + CWM	CWM	1.2g, t.i.d.	CER, LVEF, LVEDD, LVESD
Yang et al. (2019)	58/60	68/50	49.14 ± 13.52	47.89 ± 12.83	Ⅱ, Ⅲ, Ⅳ	6 months	QCC + CWM	CWM	1.2 g, t.i.d.	LVEF、LVEDD、LVESD
Wang and Chang (2019)	68/68	73/63	67.98 ± 6.03	68.54 ± 6.12	Ⅱ, Ⅲ	4 weeks	QCC + CWM	CWM	1.2g, t.i.d.	CER, LVEF, BNP
Liu and He (2019)	41/41	37/45	41.41 ± 5.35	41.39 ± 5.32	NR	2 weeks	QCC + CWM	CWM	1.2g, t.i.d.	CER, LVEF, LVEDD, LVESD, BNP, hs-CRP
Dong (2019)	40/39	51/28	53.7 ± 10.6	54.9 ± 11.2	Ⅱ, Ⅲ, Ⅳ	2 weeks	QCC + CWM	CWM	1.2 g, t.i.d.	CER, LVEF, LVEDD, LVESD, 6MWD
Dai and Wang (2019)	48/47	50/45	37.72 ± 3.85	37.86 ± 3.71	Ⅲ, Ⅳ	12 weeks	QCC + CWM	CWM	1.2 g, t.i.d.	CER, LVEF
Zhao (2018)	40/40	50/30	61.5 ± 12.3	62.2 ± 12.6	NR	4 weeks	QCC + CWM	CWM	1.2 g, t.i.d.	LVEF, LVEDD, LVESD, 6MWD, IL-6, TNF-α, HMGB1
Yao (2018)	48/48	59/37	54.60 ± 5.61	55.21 ± 4.68	Ⅱ, Ⅲ	4 weeks	QCC + CWM	CWM	1.2g, t.i.d.	CER
Wu et al. (2018)	278/278	297/259	53.19 ± 19.57	49.94 ± 21.58	Ⅱ, Ⅲ	12 months	QCC + CWM	CWM	1.2 g, t.i.d.	LVEF, LVEDD, LVESD, ARs
Wang (2018)	47/47	50/44	50.81 ± 10.61	49.12 ± 9.54	NR	3 months	QCC + CWM	CWM	1.2g, t.i.d.	CER, ARs
Hang (2018)	30/30	37/23	52.1 ± 3.2	52.2 ± 3.4	Ⅲ, Ⅳ	4 weeks	QCC + CWM	CWM	1.2 g, t.i.d.	CER, LVFE
Fang (2018)	78/78	67/99	46.22 ± 0.77	46.03 ± 0.32	Ⅱ, Ⅲ, Ⅳ	2 weeks	QCC + CWM	CWM	1.2 g, t.i.d.	CER, ARs
Jing et al. (2017)	43/42	40/45	41.3 ± 10.1	41.1 ± 9.8	NR	2 weeks	QCC + CWM	CWM	1.2g, t.i.d.	CER, LVEF, LVEDD, LVESD, 6MWD, BNP, hs-CRP
Ma X. Y et al., 2016	31/32	34/29	NR		Ⅱ, Ⅲ	6 months	QCC + CWM	CWM	1.2 g, t.i.d.	CER, LVEF
Leng (2016)	31/31	36/26	54.89 ± 8.47	55.19 ± 7.55	Ⅲ, Ⅳ	1 month	QCC + CWM	CWM	1.2 g, t.i.d.	LVEF, LVEDD, 6MWD, ARs
Han (2016)	15/15	17/13	46 ± 2.97	45 ± 3.41	Ⅱ, Ⅲ, Ⅳ	3 months	QCC + CWM	CWM	1.2 g, t.i.d.	CER
Zhou and Sun (2015)	24/20	25/19	46.45 ± 28.29	47.38 ± 25.46	Ⅱ, Ⅲ	6 months	QCC + CWM	CWM	1.2 g, t.i.d.	LVEF, LVEDD, hs-CRP, TNF-α
Wu et al. (2014)	24/24	35/13	53.9 ± 9.3	52.6 ± 10.7	Ⅱ, Ⅲ	12 weeks	QCC + CWM	CWM	1.2g, t.i.d.	LVEF, 6MWD, IL-6, TNF-α, HMGB1
Yi and Ou-yang (2014)	45/45	51/39	52.4 ± 12.7	53.1 ± 13.4	Ⅱ, Ⅲ, Ⅳ	4 weeks	QCC + CWM	CWM	1.2g, t.i.d.	LVEF, LVEDD, LVESD, BNP
Ren (2015)	25/25	31/19	55.2 ± 2.3	56.2 ± 2.4	NR	12 weeks	QCC + CWM	CWM	1.2 g, t.i.d.	LVEF, 6MWD, HMGB1
Zhang et al. (2013)	35/35	42/28	NR		NR	8 weeks	QCC + CWM	CWM	1.2 g, t.i.d.	CER, LVEF, LVEDD, LVESD, BNP
Yang et al. (2013)	34/34	40/28	53.8 ± 10.1	54.6 ± 10.8	Ⅱ, Ⅲ, Ⅳ	12 weeks	QCC + CWM	CWM	1.2 g, t.i.d.	LVEF, LVEDD, ARs
Yuan (2012)	30/32	36/26	41.65 ± 9.33	43.08 ± 7.55	Ⅲ, Ⅳ	1 month	QCC + CWM	CWM	1.2 g, t.i.d.	LVEF, LVEDD, 6MWD
Sun et al. (2012)	30/30	48/12	42.52	41.23	NR	3 months	QCC + CWM	CWM	1.2g, t.i.d.	CER
Zhu et al. (2010)	40/40	48/31	52.3 ± 14.2 (total)		Ⅱ, Ⅲ, Ⅳ	7 months	QCC + CWM	CWM	1.2g, t.i.d.	LVEF, LVEDD
Liu et al. (2009)	21/20	25/16	41 ± 11	40 ± 10	Ⅱ, Ⅲ	6 months	QCC + CWM	CWM	1.2 g, t.i.d.	CER, LVEF, LVEDD, BNP, 6MWD, ARs

T, trial group; C, control group; NR, not report; QCC, Qili Qiangxin capsule; CWM, the conventional western medicine; t.i.d., three times a day; CER, clinical efficiency rate; LVEF, left ventricular ejection fractions; LVEDD, left ventricular end-diastolic dimension; LVESD, left ventricular end-systolic diameter; BNP, brain natriuretic peptide; 6MWD, 6 min walking distance; ARs, adverse reactions; hs-CRP, hypersensitive-C-Reactive Protein; IL-6, Interleukin- 6; TNF-α, tumor necrosis factor-α; HMGB1, high mobility group protein B1.

### Literature Quality Assessment

**FIGURE 2:**
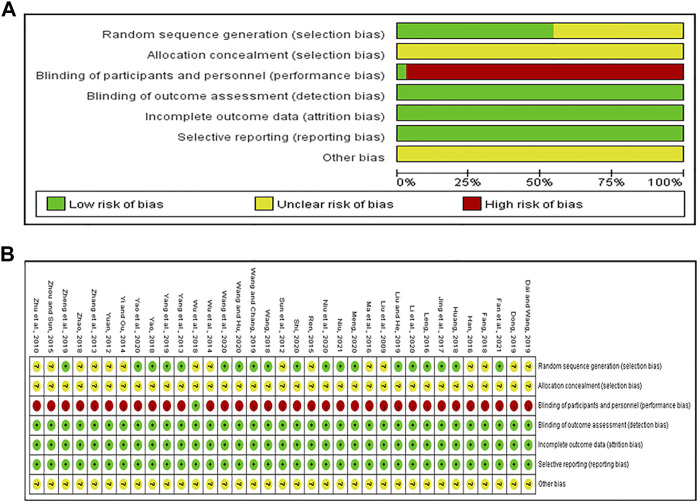
Assessment of risk of bias. **(A)** risk of bias graph and **(B)** risk of bias summary.

Nineteen RCTs specifically described methods for generating random sequences: 17 RCTs utilized the random number table (Yang et al., 2013; Leng, 2016; Jing et al., 2017; Wang, 2018; Yao, 2018; Liu and He, 2019; Wang and Chang, 2019; Yang et al., 2019; Zheng et al., 2019; Li et al., 2020; Niu et al., 2020; Shi, 2020; Wang et al., 2020; Wang and Hu, 2020; Yao et al., 2020; Fan et al., 2021; Niu, 2021), 2 RCTs utilized random lottery (Hang, 2018; Meng, 2020), which were considered to be low risk of bias. The other RCTs did not introduce the randomization method in detail and were rated as unclear risk of bias. All studies did not adequately report allocation concealment details, which was considered the unclear risk of bias. Only one trial (Wu et al., 2018) reported the use of double-blinding, and performance bias was evaluated as “low risk.” The remaining 34 studies did not provide information on blinding, so the performance bias was assessed as “high risk.” Because of the objectivity of outcome measures, the detection bias should be considered low risk of bias, regardless of whether the blinding was reported. All studies reported test indicators as planned, and there was no incomplete outcome data or selective reporting of research results. It is unclear whether there is another bias (Figure 2).

### Synthesis of Outcome

#### Clinical Efficiency Rate

**FIGURE 3:**
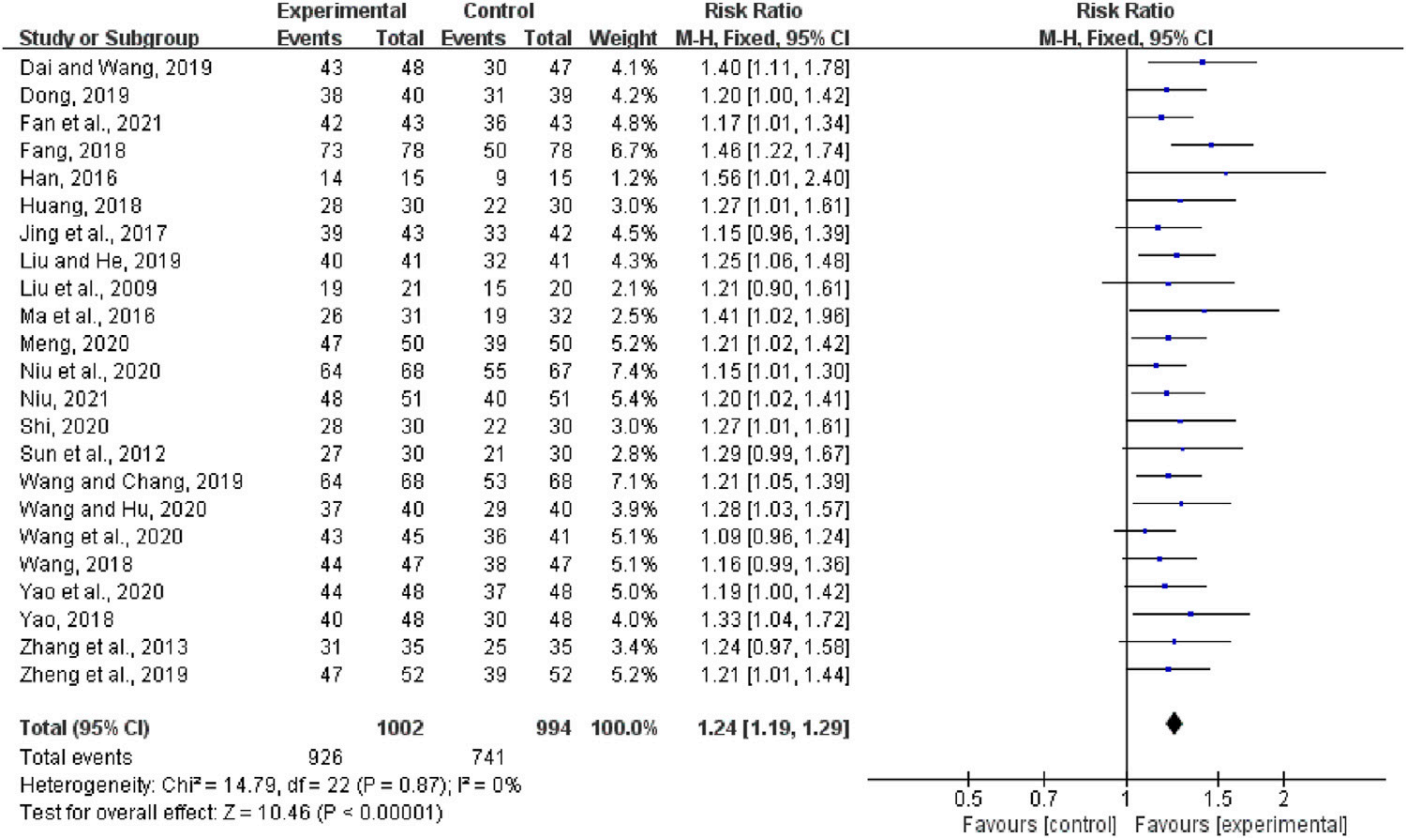
Forest plot of the clinical efficiency rate.

Twenty-three articles reported the clinical efficacy rate, including a total of 1996 patients. The heterogeneity test result suggests that the fixed-effects model should be used for Meta-analysis (*I* ^2^ = 0%, *p* = 0.87). The outcome shows that the clinical efficiency rate for the experimental group (QCC plus CWM) was significantly higher than that of CWM alone (RR = 1.24, 95% CI (1.19, 1.29), *p* < 0.00001; Figure 3).

#### LVEF

**FIGURE 4:**
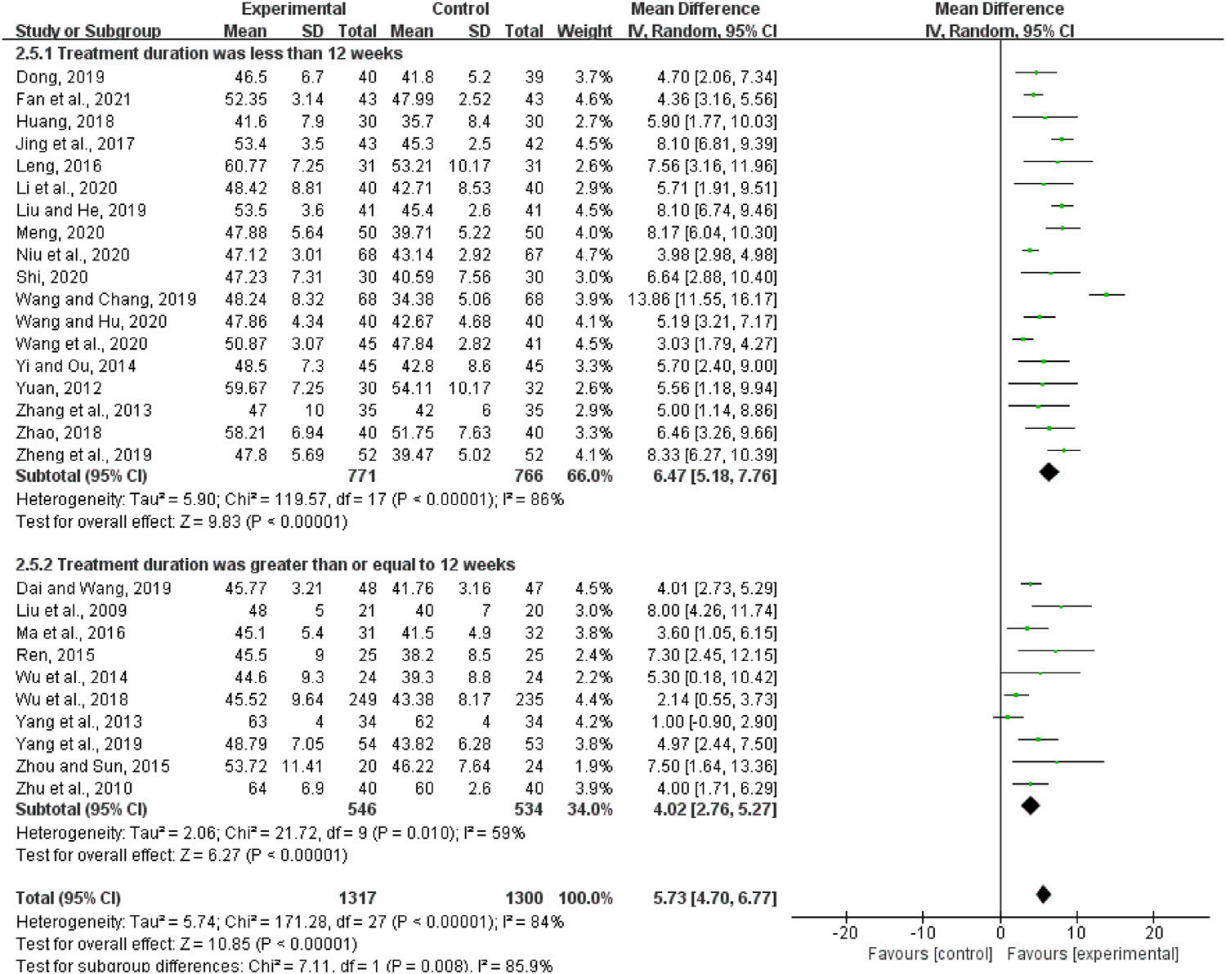
Forest plot of left ventricular ejection fraction.

Twenty-eight articles reported LVEF, including a total of 2,617 patients. The heterogeneity test result suggests that the random-effects model should be used for meta-analysis (*I* ^2^ = 84%, *p* < 0.00001). The results showed that the experimental group had significant advantages in ameliorating LVEF over the control group (MD = 5.73, 95%CI (4.70, 6.77), *p* < 0.00001; Figure 4).

According to the treatment duration, twenty-eight trials were subdivided into two subgroups (less than 12 weeks subgroup and more than or equal to 12 weeks subgroup). There was relatively high heterogeneity in each subgroup (less than 12 weeks: *I* ^2^ = 86%, *p* < 0.00001; more than or equal to 12 weeks: *I* ^2^ = 59%, *p* = 0.01). The random-effects model was used. According to Figure 4, when the treatment duration was less than 12 weeks, the LVEF in the experimental group was higher than that in the control group (MD = 6.47, 95%CI (5.18, 7.76), *p* < 0.00001). When the treatment duration was more than or equal to 12 weeks, the LVEF in the experimental group was also higher than that in the control group (MD = 4.03, 95%CI (2.76, 5.27), *p* < 0.00001). The result of subgroup analysis was limited by substantial heterogeneity. More trials with good homogeneity would be needed to demonstrate the results.

#### LVEDD

A total of twenty studies pertaining to 1970 patients reported LVEDD. The heterogeneity test results showed that the random-effects model should be used (*I* ^2^ = 66%, *p* < 0.0001). The results showed that compared with the control group, the experimental group had more advantages in the treatment of LVEDD in DCM patients (MD = −4.09, 95%CI (−4.91, −3.27), *p* < 0.00001).

**FIGURE 5:**
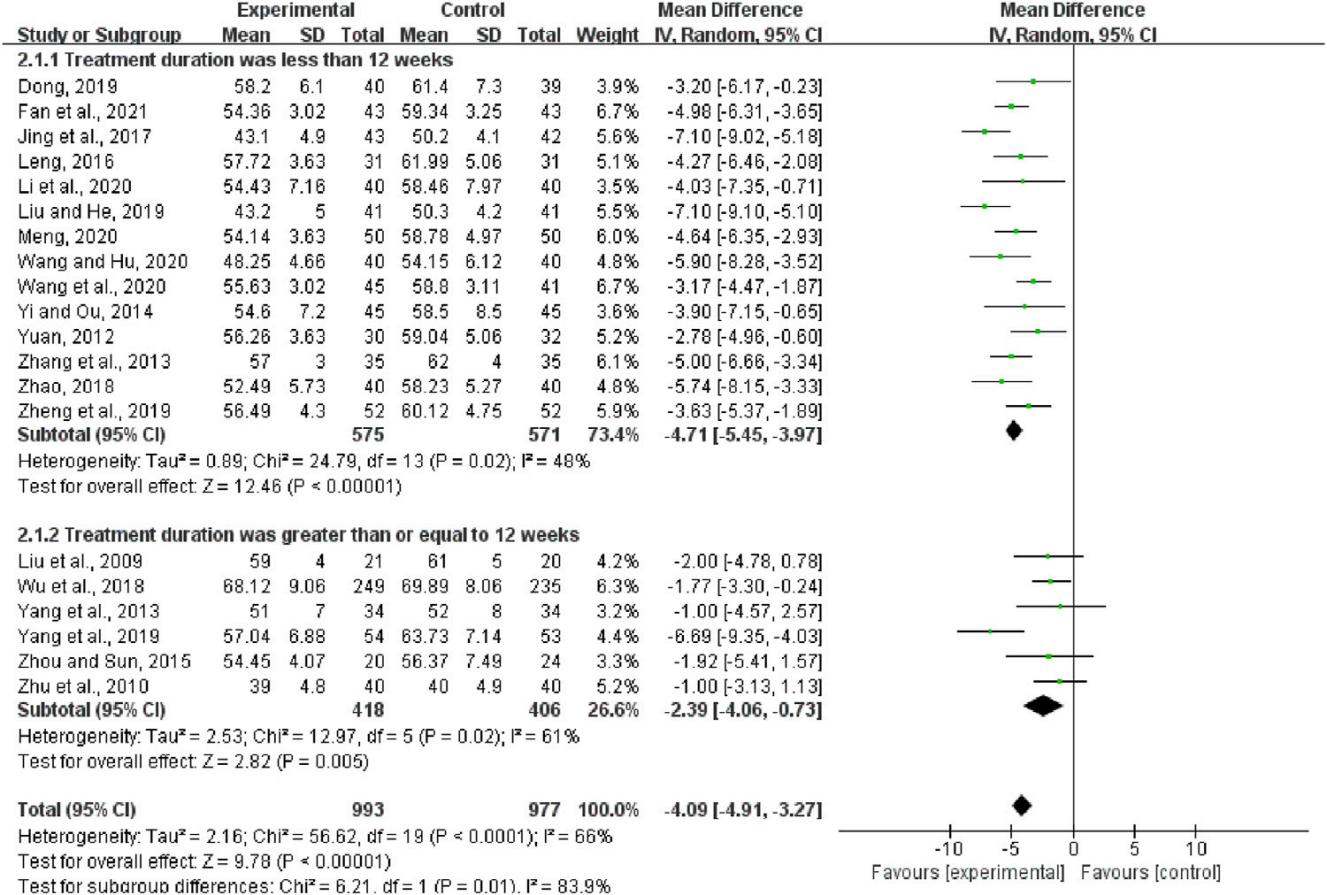
Forest plot of left ventricular end-diastolic dimension.

According to the treatment duration, they were subdivided into two subgroups (less than 12 weeks subgroup and more than or equal to 12 weeks subgroup). There was low heterogeneity in each subgroup (less than 12 weeks: *I* ^2^ = 48%, *p* = 0.02; more than or equal to 12 weeks: *I* ^2^ = 61%, *p* = 0.02). The random-effects model was used. According to Figure 5, when the treatment duration was less than 12 weeks, the LVEDD in the experimental group was lower than that in the control group (MD = −4.71, 95%CI (−5.45, −3.97), *p* < 0.00001). When the treatment duration was more than or equal to 12 weeks, the LVEDD in the experimental group was also lower than that in the control group (MD = −2.39, 95%CI (−4.06, −0.73), *p* = 0.005). The results of subgroup analysis indicated that the treatment duration might be the source of heterogeneity.

#### LVESD

**FIGURE 6:**
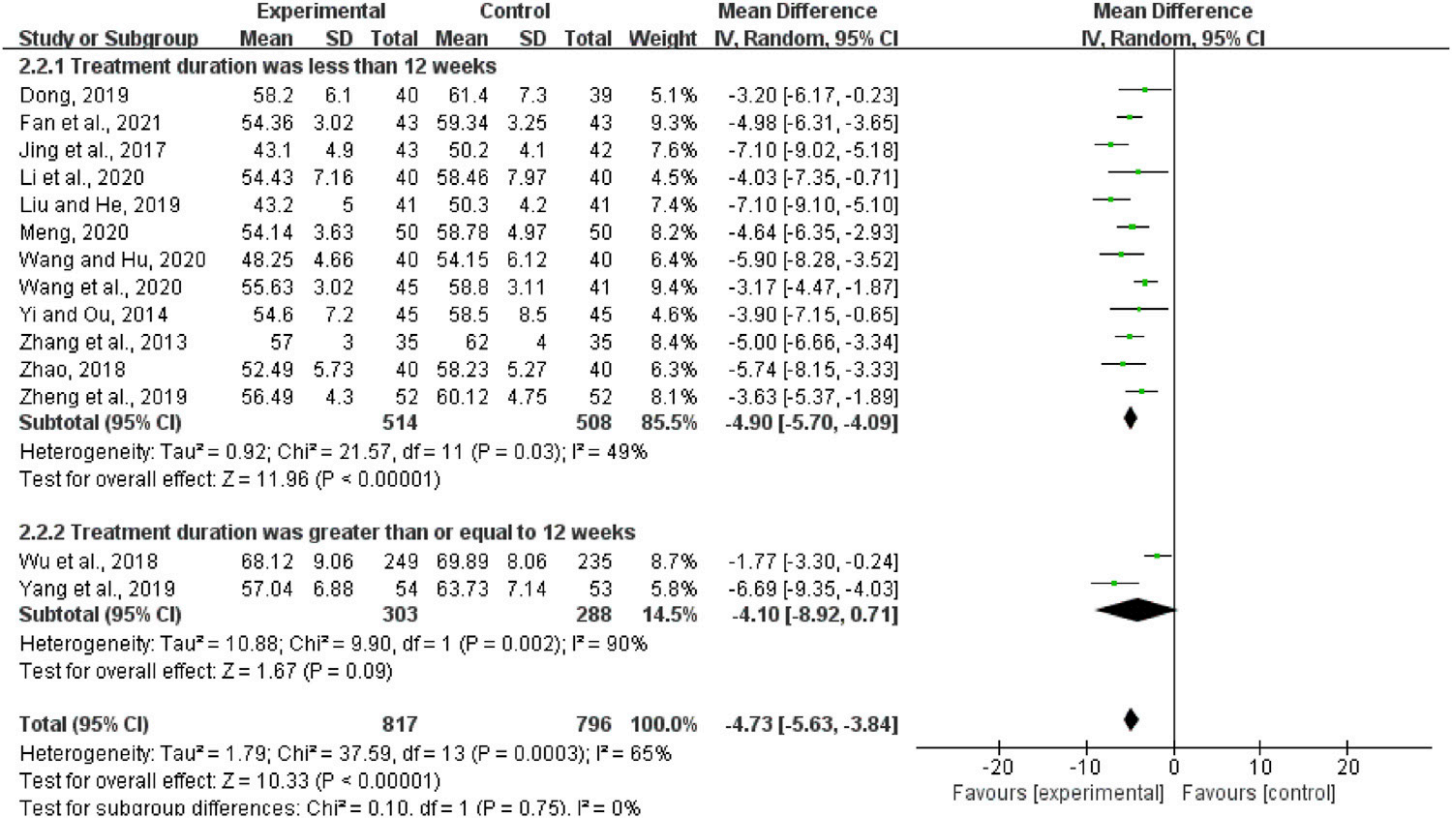
Forest plot of left ventricular end-systolic diameter.

Fourteen trials with 1,613 patients reported the treatment effects on LVESD. The results demonstrated that QCC combined with CWM showed a weighty decrease in the LVESD level compared with CWM alone (MD = -4.73, 95%CI (-5.63, -3.84), *p* < 0.00001; Figure 6). However, significant heterogeneity was identified among the studies (*I* ^2^ = 65%, *p* = 0.0003).

According to the treatment duration, they were subdivided into two subgroups (less than 12 weeks subgroup and more than or equal to 12 weeks subgroup). There was moderate heterogeneity in each subgroup (less than 12 weeks: *I* ^2^ = 49%, *p* = 0.03; more than or equal to 12 weeks: *I* ^2^ = 90%, *p* = 0.002). The random-effects model was used. As shown in Figure 6, the LVESD in the experimental group was lower than that in the control group when the treatment duration was less than 12 weeks (MD = −4.90, 95%CI (−5.70, −4.09), *p* < 0.00001). When the treatment duration was more than or equal to 12 weeks, the LVESD in the experimental group was also lower than that in the control group (MD = 4.03, 95%CI (2.76, 5.27), *p* < 0.00001).

#### BNP

**FIGURE 7:**
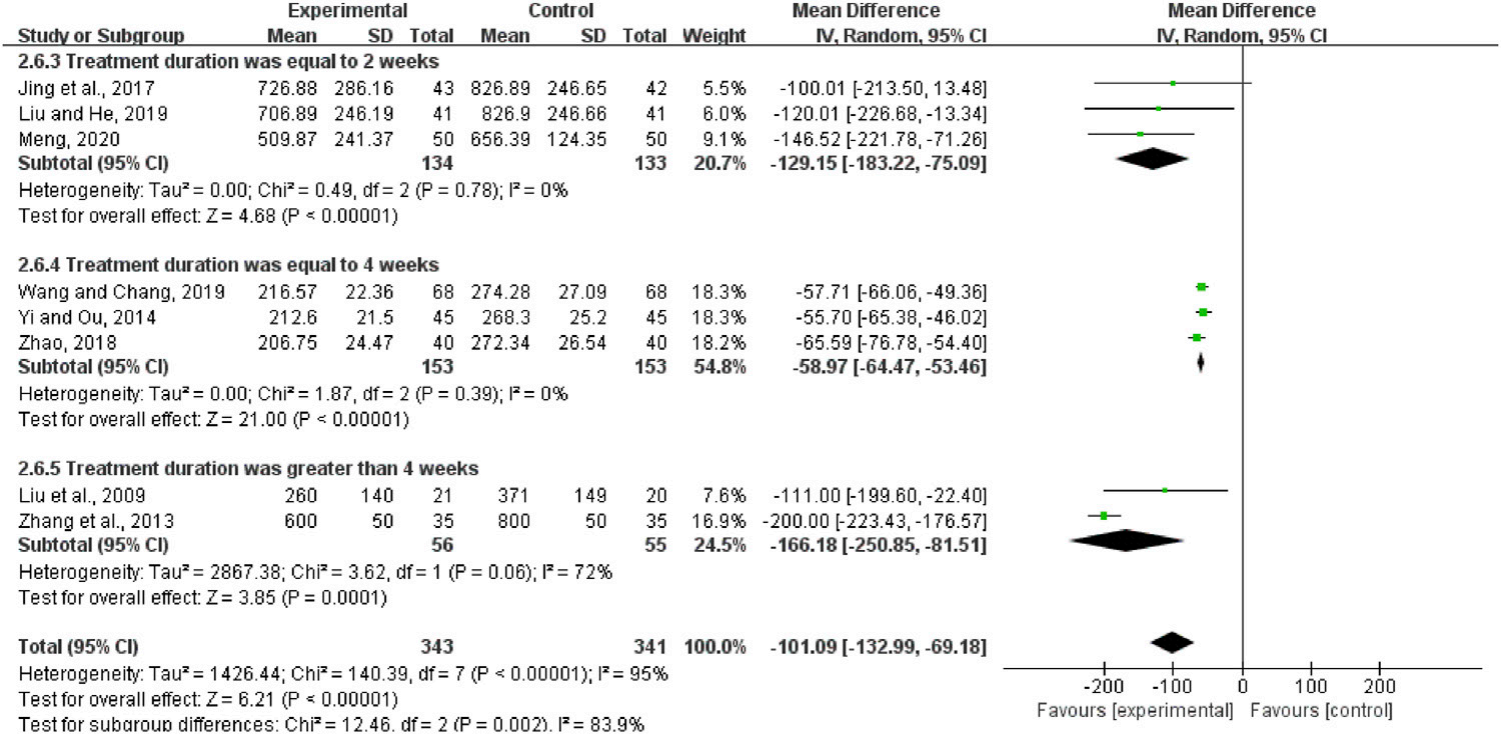
Forest plot of brain natriuretic peptide.

Eight studies with 684 patients reported the treatment effects on BNP. We use the random-effects model for statistical analysis based on the heterogeneity (*I* ^2^ = 95%, *p* < 0.00001). The results showed that the experimental group was significantly better than the control group in ameliorating the BNP of DCM patients (MD = −101.09, 95%CI (−132.99, −69.18), *p* < 0.00001; Figure 7).

According to the treatment duration, eight studies were subdivided into three subgroups (equal to 2 weeks subgroup, equal to 4 weeks subgroup, and more than 4 weeks subgroup). There was low heterogeneity in each subgroup (equal to 2 weeks: *I* ^2^ = 0%, *p* = 0.78; equal to 2 weeks: *I* ^2^ = 0%, *p* = 0.39; more than 4 weeks: *I* ^2^ = 72%, *p* = 0.06). The random-effects model was used. According to Figure 7, when the treatment duration was equal to 2 weeks, the experimental group was significantly better than the control group in improving the BNP (MD = −129.15, 95%CI (−183.22, −75.09), *p* < 0.00001). When the treatment duration was equal to 4 weeks, the experimental group was significantly better than the control group in improving the BNP (MD = -58.97, 95%CI (−64.47, −53.46), *p* < 0.00001). When the treatment duration was more than 4 weeks, the BNP in the experimental group was also lower than that in the control group (MD = −66.18, 95%CI (−250.85, −81.51), *p* = 0.0001). The results of subgroup analysis indicated that the treatment duration might be the source of heterogeneity.

#### 6MWD

**FIGURE 8:**
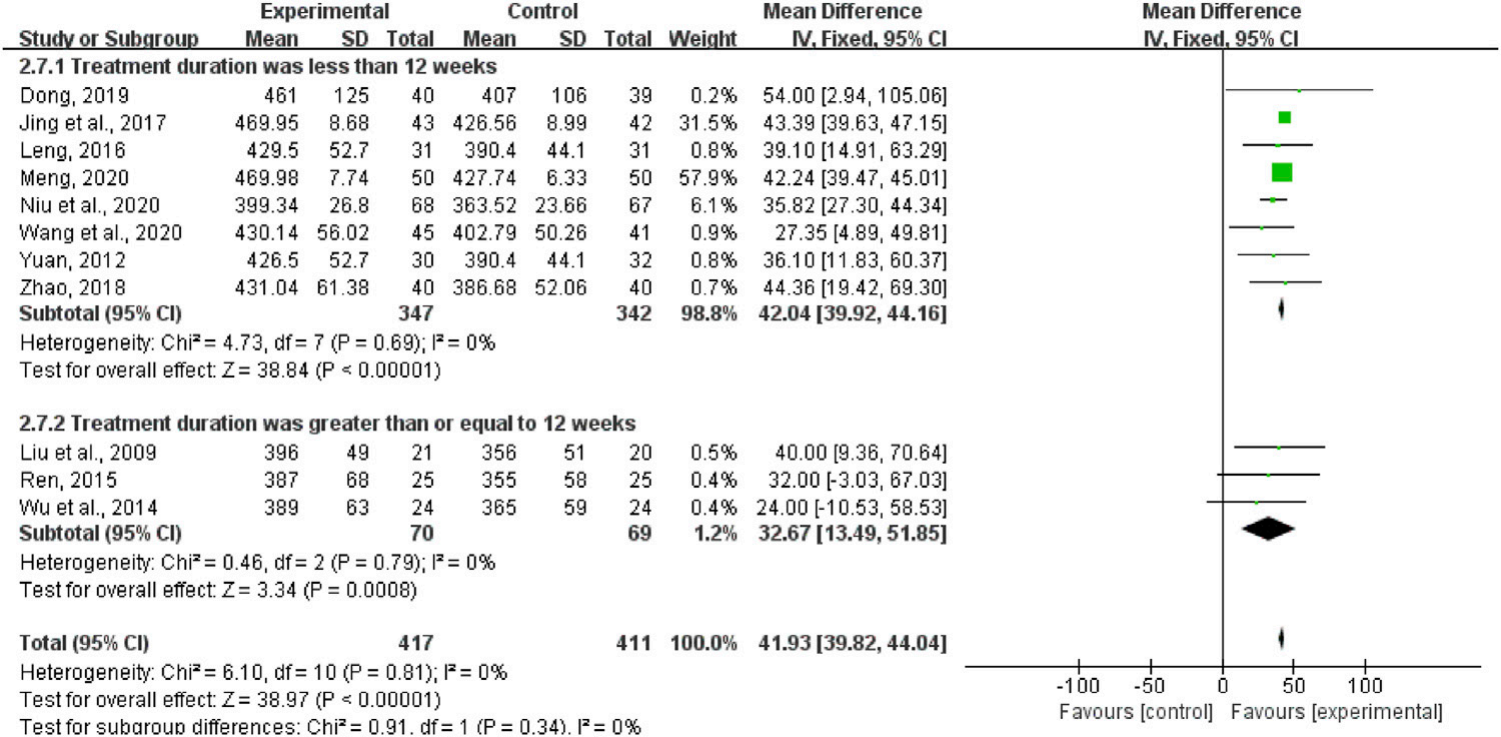
Forest plot of 6 min walking distance.

A total of 828 patients from eleven trials reported on 6MWD. The fixed-effects model was used for statistical analysis (*I* ^2^ = 0%, *p* = 0.81). The results showed that the experimental group was significantly better than the control group in improving 6MWD in DCM patients (MD = 41.93, 95%CI (39.82, 44.04), *p* < 0.00001; Figure 8).

We performed a subgroup analysis according to the treatment duration and selected fixed-effects model (less than 12 weeks: *I* ^2^ = 0%, *p* = 0.69; more than or equal to 12 weeks: *I* ^2^ = 0%, *p* = 0.79). As shown in Figure 8, the experiment group had significant advantages in ameliorating the 6MWD than the control group when the treatment duration was less than 12 weeks (MD = 42.04, 95%CI (39.92, 44.16), *p* < 0.00001). When the treatment duration was more than or equal to 12 weeks, the experiment group also had significant advantages in ameliorating the 6MWD over the control group (MD = 32.67, 95%CI (13.49, 51.85), *p* = 0.0008).

#### Hs-CRP

**FIGURE 9:**
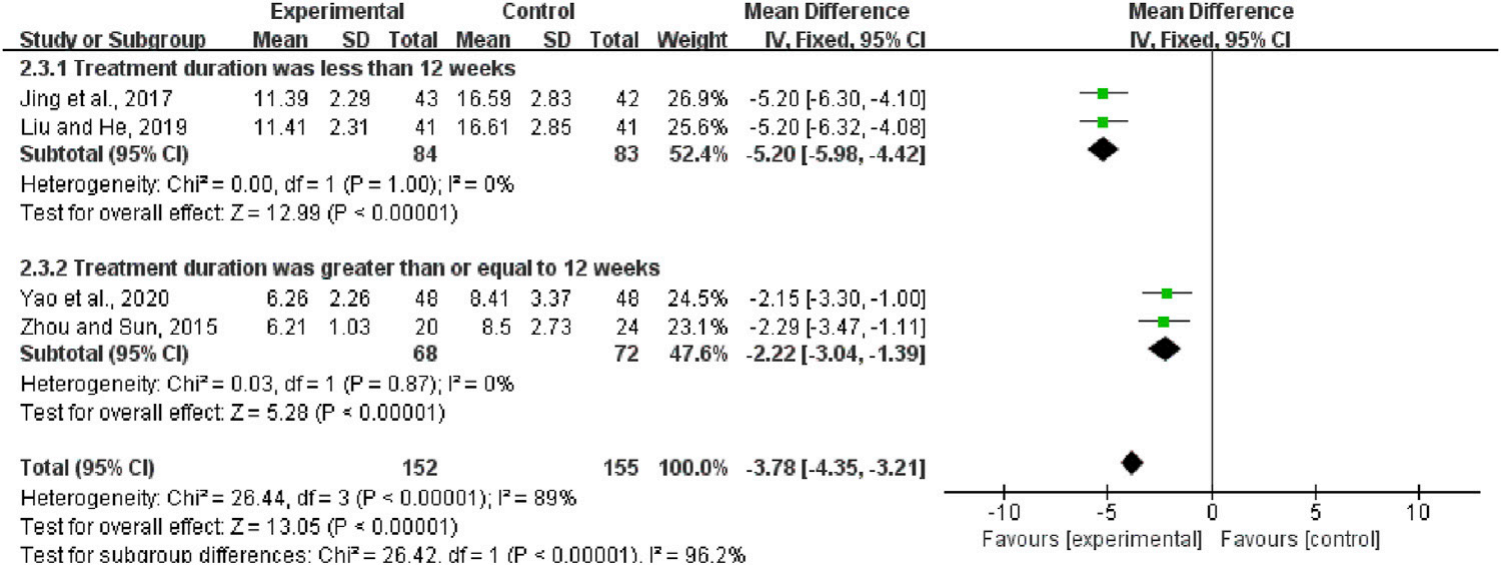
Forest plot of hypersensitive-C-Reactive Protein.

Four trials, including 307 patients, reported the treatment effects on hs-CRP. The heterogeneity test results showed that the random-effects model should be used (*I* ^2^ = 89%, *p* < 0.00001). Compared with CWM alone, QCC could significantly reduce the hs-CRP levels (MD = −3.78, 95%CI (−4.35, −3.21), *p* < 0.00001). We performed subgroup analysis according to the treatment duration. There was low heterogeneity in each subgroup (less than 12 weeks: *I* ^2^ = 0%, *p* = 1.00; more than or equal to 12 weeks: *I* ^2^ = 0%, *p* = 0.87) among the RCTs, the fixed-effects model was used. According to Figure 9, when the treatment duration was less than 12 weeks, the hs-CRP in the experimental group was lower than that in the control group (MD = −5.20, 95%CI (−5.98, −4.42), *p* < 0.00001). When the treatment duration was more than or equal to 12 weeks, the hs-CRP in the experimental group was also lower than that in the control group (MD = −2.22, 95%CI (−3.04, −1.39), *p* < 0.00001). The results of subgroup analysis indicated that the treatment duration might be the source of heterogeneity.

#### IL-6, TNF-α, and HMGB1

**FIGURE 10:**
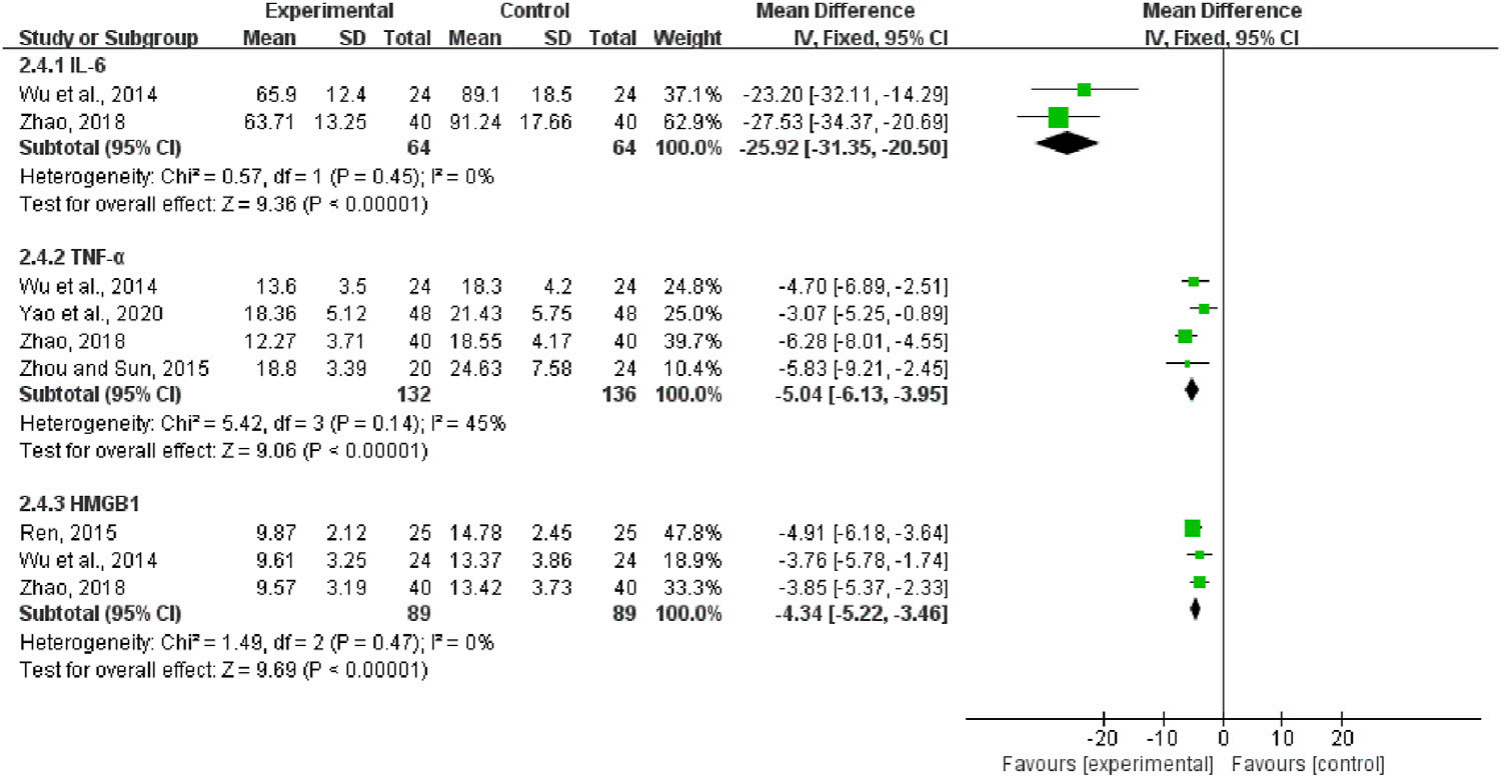
Forest plots of Interleukin- 6, tumor necrosis factor-α, and high mobility group protein B1.

Two trials, including 128 patients, reported the treatment effects on IL-6. We found that its heterogeneity was low (*I* ^2^ = 0%, *p* = 0.45). We selected a fixed-effect model. The results showed that compared with CWM alone, QCC could significantly reduce the IL-6 levels (MD = −25.92, 95%CI (−31.35, −20.50), *p* < 0.00001, Figure 10). Four trials contained 268 patients reported TNF-α, and the fixed-effect model was used for analysis (*I* ^2^ = 45%, *p* = 0.14; Figure 10). MD = -5.04, 95%CI (-6.13, -3.95), *p* < 0.00001, means that the experimental group had an advantage in ameliorating the TNF-α of patients with DCM. A total of 178 patients from three trials reported on HMGB1. The fixed-effect model was used for statistical analysis (*I* ^2^ = 0%, *p* = 0.47). The results showed that the experimental group was significantly better than the control group in improving HMGB1 in DCM patients (MD = -4.34, 95%CI (−5.22, −3.46), *p* < 0.00001; Figure 10).

#### ARs

**FIGURE 11:**
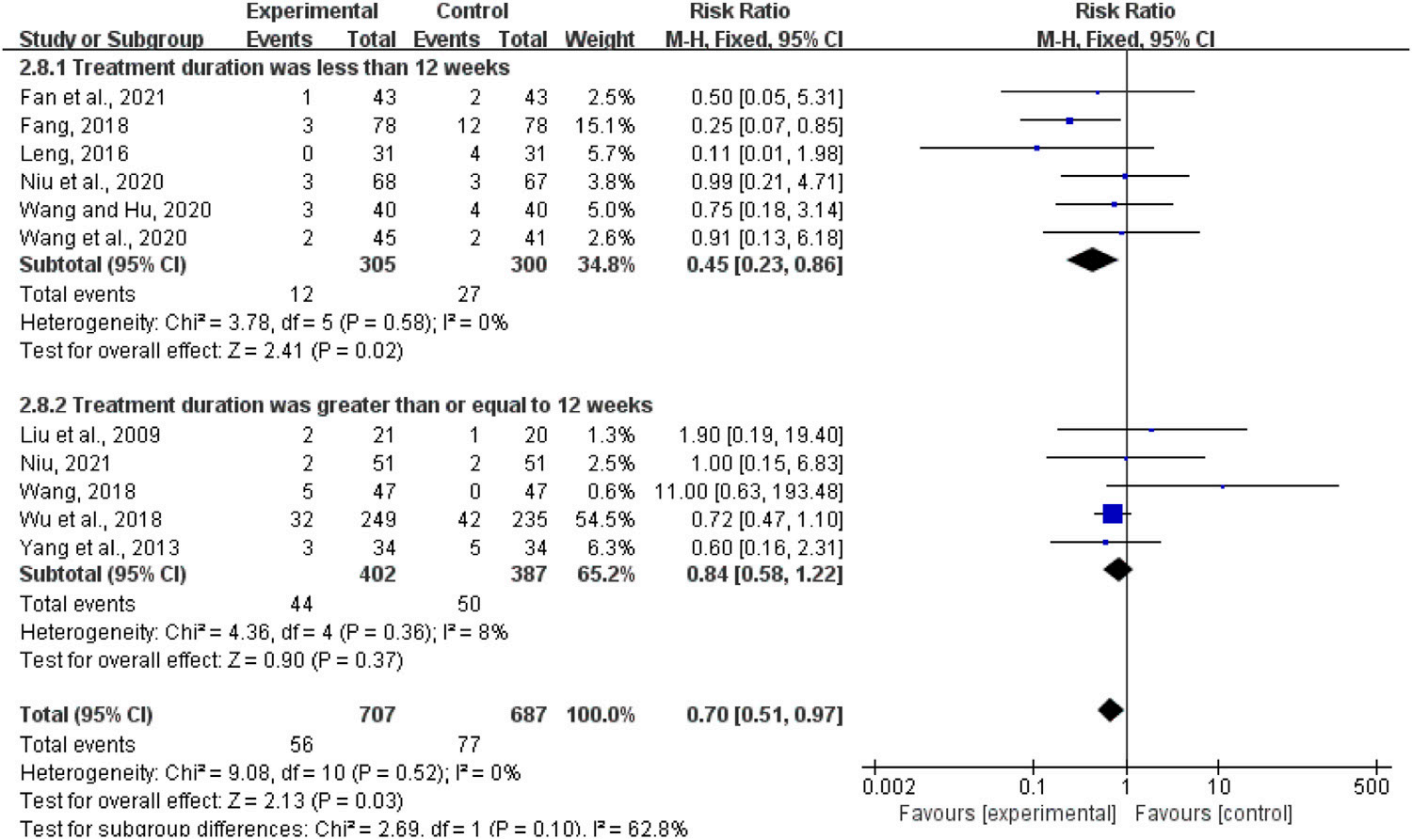
Forest plot of adverse reactions.

Eleven studies with a total of 1,394 patients reported ARs, included dizziness (Wang et al., 2020; Wang and Hu, 2020; Wu et al., 2018), headache (Fang, 2018; Wang, 2018; Wu et al., 2018; Niu et al., 2020; Niu, 2021), hypotension (Wang et al., 2020; Fan et al., 2021), fatigue (Fang, 2018; Niu et al., 2020), dry cough (Liu et al., 2009; Yang et al., 2013; Wu et al., 2018; Wang et al., 2020; Fan et al., 2021), palpitations (Yang et al., 2013; Leng, 2016; Niu et al., 2020; Wang et al., 2020; Wang and Hu, 2020; Fan et al., 2021), and gastrointestinal adverse reactions such as nausea and vomiting (Liu et al., 2009; Yang et al., 2013; Leng, 2016; Fang, 2018; Wang, 2018; Wu et al., 2018; Wang et al., 2020; Wang and Hu, 2020; Niu, 2021). Moreover, the fixed-effects model was used for statistical analysis (*I* ^2^ = 0%, *p* = 0.47). The results showed that the experimental group had advantages over the control group in reducing adverse reactions (RR = 0.70, 95%CI (0.51, 0.97), *p* = 0.03; Figure 11). We performed a subgroup analysis and selected fixed-effects model (less than 12 weeks: *I* ^2^ = 0%, *p* = 0.58; more than or equal to 12 weeks: *I* ^2^ = 8%, *p* = 0.79). As shown in Figure 11, the experimental group had advantages over the control group in reducing ARs when the treatment duration was less than 12 weeks [RR = 0.45, 95%CI (0.23, 0.86), *p* = 0.02]. When the treatment duration was more than or equal to 12 weeks, the experimental group had no advantage over the control group in reducing adverse events (MD = 0.84, 95%CI (0.58, 1.22), *p* = 0.37).

### Evaluation of Publication Bias

**FIGURE 12:**
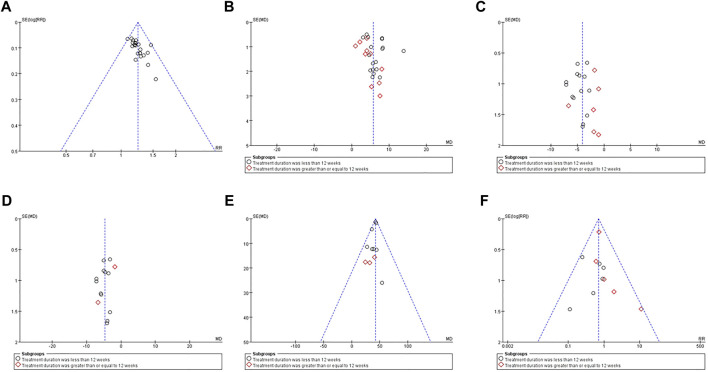
Funnel plots of between QCC combined with conventional western medicine and conventional western medicine alone for the clinical efficiency rate **(A)**, LVEF **(B)**, LVEDD **(C)**, LVESD **(D)**, 6MWD **(E)**, and ARs **(F)**.

We used the funnel plots (Figure 12) and Egger’s test (Supplementary Figure S1) to examine the possible publication bias for the outcomes of the clinical efficiency rate, LVEF, LVEDD, LVESD, 6MWD, and ARs in this meta-analysis. Consequently, the symmetrical shape of the funnel plots, as well as the *p*-values from Egger’s tests, revealed that there was no remarkable publication bias for LVEF, LVEDD, LVESD, 6MWD, and ARs (*p* = 0.225; *p* = 0.799; *p* = 0.213; *p* = 0.079; *p* = 0.635) and there may be publication bias for the clinical efficiency rate (*p* < 0.001).

### GRADE Assessment

The GRADE approach was employed to explore the quality of evidence for the six outcomes, which exhibited moderate or low quality with severe methodological problems and a heterogeneity problem. Table 3 presents an overview of the GRADE evidence.

**TABLE 3:** GRADE evidence profile.

Quality assessment			Indirectness	Imprecision	Other considerations	No of patients		RR/MD (95% CI)	Quality	Importance
No. of studies	Risk of bias	Inconsistency				QCC combined with CWM	CWM			
Clinical efficiency rate										
23	serious^a^	no serious inconsistency	no serious indirectness	no serious imprecision	Reporting bias^b^	926/1,002 (92.4%)	741/994 (74.5%)	RR = 1.24 (1.19, 1.29)	⊕⊕OO low	CRITICAL
Left ventricular ejection fraction										
28	serious^a^	serious^c^	no serious indirectness	no serious imprecision	none	1,317	1,300	MD = 5.73 (4.70, 6.77)	⊕⊕OO low	CRITICAL
Left ventricular end-diastolic dimension										
20	serious^a^	no serious inconsistency	no serious indirectness	no serious imprecision	none	993	977	MD = -4.09 (-4.91, -3.27)	⊕⊕⊕O moderate	CRITICAL
Left ventricular end-systolic diameter										
14	serious^a^	no serious inconsistency	no serious indirectness	no serious imprecision	none	817	796	MD = -4.73 (-5.63, -3.84)	⊕⊕⊕O moderate	CRITICAL
Adverse reactions										
11	serious^a^	no serious inconsistency	no serious indirectness	no serious imprecision	none	56/707 (7.9%)	76/687 (11.1%)	RR = 0.70 (0.51, 0.97)	⊕⊕⊕O moderate	CRITICAL
Brain natriuretic peptide										
8	serious^a^	no serious inconsistency	no serious indirectness	no serious imprecision	none	343	341	MD = -101.09 (-132.99, -69.18)	⊕⊕⊕O moderate	IMPORTANT
6 min walking distance										
11	serious^a^	no serious inconsistency	no serious indirectness	no serious imprecision	none	417	411	MD = 41.93 (39.82, 44.04)	⊕⊕⊕O moderate	IMPORTANT
Hypersensitive-C-Reactive Protein										
4	serious^a^	no serious inconsistency	no serious indirectness	no serious imprecision	none	152	155	MD = -3.78 (-4.35, -3.21)	⊕⊕⊕O moderate	IMPORTANT
Interleukin- 6										
2	serious^a^	no serious inconsistency	no serious indirectness	no serious imprecision	none	64	64	MD = -25.92 (-31.35, -20.50)	⊕⊕⊕O moderate	IMPORTANT
Tumor necrosis factor-α										
4	serious^a^	no serious inconsistency	no serious indirectness	no serious imprecision	none	132	136	MD = -5.04 (-6.13, -3.95)	⊕⊕⊕O moderate	IMPORTANT
High mobility group protein B1										
3	serious^a^	no serious inconsistency	no serious indirectness	no serious imprecision	none	89	89	MD = -4.34 (-5.22, -3.46)	⊕⊕⊕O moderate	IMPORTANT

QCC, Qili Qiangxin capsule; CWM, the conventional western medicine; CI, confidence interval; MD, mean difference; RR, risk ratio.

a Random protocol, blinding, and allocation concealment of some studies were not clear.

b Quantitative evaluation of the included data indicated publication bias.

c Heterogeneity (*I* ^2^ > 50%, *p* < 0.05) was found.

## Discussion

Generally, DCM patients present with typical signs of heart failure (progressive dyspnoea, fatigue, physical exertion, ankle swelling, orthopnea, mood disorders), ventricular arrhythmia, thromboembolic events, and sudden cardiogenic death (Prasad and Halliday, 2021). Patients have a 1-year survival rate of 70%–75% without aggressive treatment and a 5-years survival rate as low as 50% (Reichart et al., 2019). Based on the original effective neuroendocrine suppression, the role of drugs in further reducing the mortality and disability rate is more and more limited, while non-drug treatment is challenging to promote due to issues including technology, cost, and indication. Thus, it is essential to develop new therapies to treat patients with DCM. Integrated traditional Chinese and Western medicine has been developed as a novel therapeutic method for DCM treatment. It has the unique advantage of improving clinical symptoms and reducing the ARs compared with CWM alone. However, no meta-analysis and systematic review on the efficacy and safety of QCC combined with CWM in DCM treatment has been established. Therefore, we performed this study to systematically evaluate the safety and effectiveness of QQC in the treatment of DCM.

### Summary of Evidence

Through the statistical analysis of a total of 3,334 patients in the 35 included studies, QQC combined with CWM had advantages in the treatment of DCM, which is reflected in the clinical efficiency rate, the improvement of 6-MWT, LVEF, LVEDD, LVESD, and BNP, and the difference had statistical advantages. The clinical efficiency rate is the sum of the percentage of cardiac function in patients who achieve a complete or partial response. The clinical efficiency rate in patients who had been treated with QCC plus CWM was 92.4% (926/1002). The clinical efficiency rate in patients who had been treated with CWM alone was 74.5% (741/994). The results indicated that the clinical efficiency rate of QCC combined with CWM was remarkably higher than CWM independently. Ultrasound cardiogram (UCG) is a robust test method for cardiac function or structure assessment in diagnosing and evaluating dilated cardiomyopathy. UCG can detect ventricular systolic and diastolic function, wall motion, and mural thrombus. Many experiments show that left ventricular dysfunction is closely related to the prognosis of DCM patients (Sreenivasan and Jain, 2021). Parameters like LVEF, LVEDD, and LVESD reflect left ventricular function and size and provide diagnostic and prognostic value for patients. 6MWD, a good indicator of exercise tolerance, asks the patient to walk as fast as possible in a straight corridor and measures the walking distance of 6 min 6MWD clearly shows improvement in exercise tolerance in patients before and after treatment. BNP testing is recommended for screening, diagnosis and differential diagnosis, and assessment of disease severity and prognosis (Halliday et al., 2019). In the present study, 28 articles with 2,617 patients reported LVEF, in which the experimental group had significant advantages in ameliorating LVEF over the control group. Nevertheless, the result was limited by unidentified heterogeneity. Limited by the existence of substantial heterogeneity, it is necessary to increase the sample size to verify whether the use of QCC in patients with dilated cardiomyopathy actually increases LVEF levels.

Meta-analysis in this study proves that QCC could remarkably reduce the levels of hs-CRP, IL-6, TNF-α, and HMGB1 in DCM patients. IL-6, TNF-α, and other inflammatory markers are prevalent in patients with DCM, especially in patients with disease progression, and inflammatory markers levels are directly correlated with cardiac function levels (Anzai, 2018). HMGB1, which serves as an early promoter and facilitator of inflammation, can cause various inflammatory responses, tissue regeneration, and heart failure. Clinical studies have shown that HMGB1 and its associated inflammatory cytokines (IL-6, TNF-α) may be involved in the pathophysiological processes of dilated cardiomyopathy and accelerated heart function decline (Wu et al., 2013).

In this study, eleven articles with 1,394 patients were observed for the incidence of ARs. The incidence of ARs in patients who had been treated with QCC plus CWM was 7.5% (56/707). The incidence of ARs in patients who had been treated with CWM solely was 11.1% (76/687). The result concluded that a significantly lower incidence rate of ARs in the experimental group compared with the control group suggested that adding the use of QCC combined with CWM was safe. Hence, we provide supporting evidence that, to a remarkable extent, QCC can potentially be recommended for planned use for DCM patients. However, in our meta-analysis, LVEDD and hs-CRP showed high heterogeneity, and we identified sources of heterogeneity through subgroup analysis. The results of subgroup analysis indicated that the treatment duration might be the source of heterogeneity.

### Comparison With Previous Studies

Multiple meta-analyses and systematic reviews have confirmed the effectiveness and safety of traditional Chinese medicine (TCM) in treating DCM. A meta-analysis constituting 21 RCTs involving 1,566 patients revealed that oral Chinese herbal medicine effectively improves overall efficacy, LVEF, LVEDD, stroke volume (SV), BNP, and 6MWD in the treatment of DCM (Zhu et al., 2016), which was consistent with our results. However, BNP, LVEF, SV, and 6MWD represented high heterogeneity in their meta-analysis. The reason may be numerous varieties of Chinese herbal compounds and control groups, insufficient clinical samples, and follow-up time. The findings of another systematic review and meta-analysis indicated that five Chinese medicine injections significantly improved performance compared with CWM solely in treating DCM (Cao et al., 2022). This study suggested that the clinical selection of Chinese medicine injections should consider the individual patient’s circumstances, as different types of Chinese medicine injections have different advantages. Besides, a randomized controlled trial confirmed that QCC treatment showed superior performance compared to the placebo concerning cardiac function classification, LVEF, 6MWD, and quality of life (Li et al., 2013).

### Limitations

This study has the following limitations: 19 of the 35 studies described the specific randomization method adequately, accounting for 54.28%, while the rest only mentioned randomization and did not specifically describe the randomization method, and all did not mention the use of allocation concealment, thus potentially creating a risk of bias in the study. At the same time, some of the included studies had small sample sizes, with a total of 14 studies with a sample size of less than 80, accounting for 40%, which makes the results of the included studies unreliable. The efficacy measures lacked endpoint event measures, such as the incidence rate of cardiovascular events, and there was little safety data. The occurrence of adverse reactions was reported only in seven studies. The use of conventional western medicine was not the same in all studies. The span of treatment courses was large in different studies (for a minimum of 2 weeks or up to 12 months), which may lead to an increase in clinical heterogeneity. None of the 35 included studies mentioned the sample size calculation method, reflecting the lack of rigorous sample size estimation, resulting in less reliable study results.

### Implications for Research

Firstly, it is evident that strategies that improve the methodological quality of RCTs are urgently needed. Going forward, we recommend establishing and reporting the randomized controlled trial of QCC strictly under the CONSORT 2010 statement (Cheng et al., 2017), clearly implementing allocation concealment and blinding, clarify the specific randomization method, record the cases lost to follow-up in detail, and strengthen quality control. Secondly, we propose to establish a curative effect evaluation system that conforms to the characteristics of traditional Chinese medicine, excavate sensitive and practical indicators of TCM, and highlight the advantages of TCM. Thirdly, the safety of QCC for dilated cardiomyopathy needs further investigation. We suggest improving reporting of adverse events and adverse drug reactions related to Qili Qiangxin Capsules according to the standard reporting format (Bian et al., 2010). Fourthly, the overall quality of evidence was graded as low or moderate in our study. Consistent recommendations can be reached through a rigorous expert consensus method in the absence of high-level, evidence-based findings. We propose to revise TCM clinical practice guidelines of QCC combined with CWM for DCM based on the formed expert consensus.

## Conclusion

In summary, the available evidence shows that Qili Qiangxin Capsule combined with conventional western medicine can significantly improve the clinical efficacy rate, LVEF, LVEDD, LVESD, BNP, and 6-MWT, decrease the levels of hs-CRP, IL-6, TNF-α, and HMGB1 in DCM patients, and reduce the occurrence of adverse reactions. However, due to some limitations in the methodology of the included trials, high-quality RCT studies with more samples, scientific design, multiple centers and strict implementation are still needed to further demonstrate this conclusion in the future, providing higher quality evidence support for the application of Qili Qiangxin Capsule in clinical practice.

## Data Availability

The original contributions presented in the study are included in the article/Supplementary Material, further inquiries can be directed to the corresponding authors.
