# Supplementary material for: Efficacy and Safety of Qili Qiangxin Capsule on Dilated Cardiomyopathy: A Systematic Review and Meta-Analysis of 35 Randomized Controlled Trials
Source: Front Pharmacol. 2022 Apr 28;13:893602. doi: 10.3389/fphar.2022.893602 (PMC9095857; doi:10.3389/fphar.2022.893602)
Supplement: Supplementary file 1 [file DataSheet1.ZIP › Figure S1.Egger’s test.docx]

Supplementary Material

**Figure S1.** Egger’s test of the clinical efficiency rate (A), LVEF(B), LVEDD(C), LVESD(D), 6MWD(E), and ARs(F).


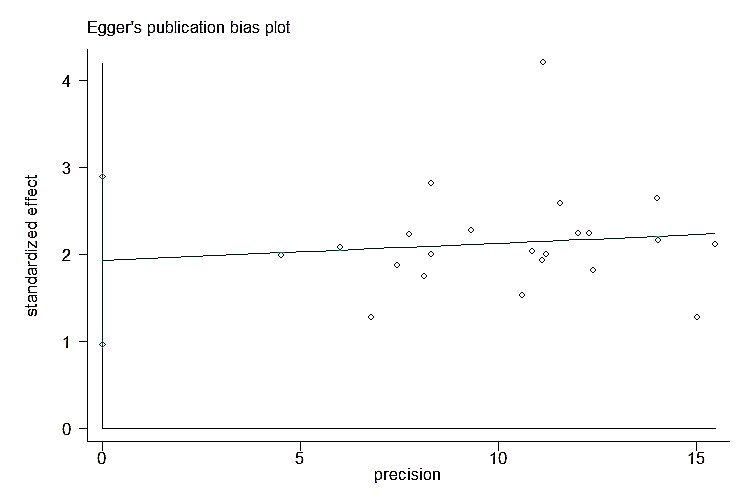


**A.** The Egger’s test of the clinical efficiency rate


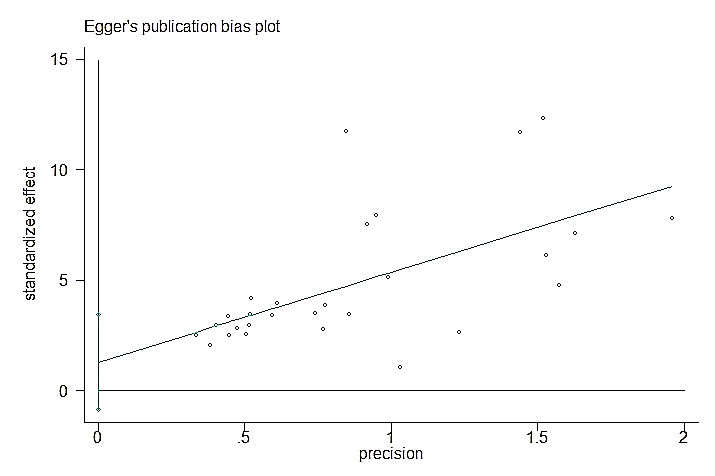


**B.** The Egger’s test of LVEF


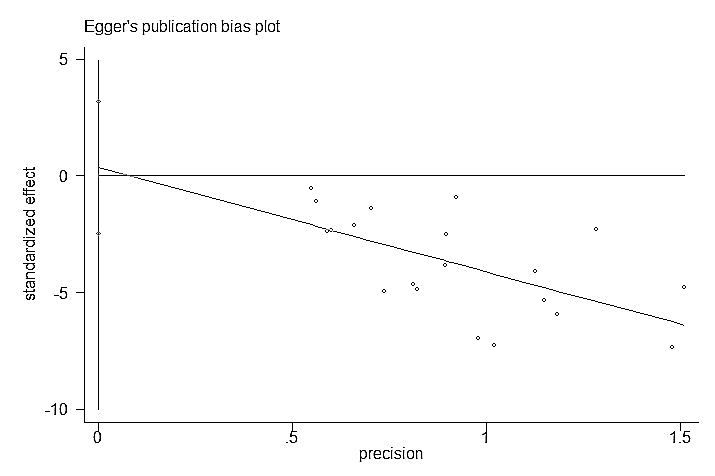


**C.** The Egger’s test of LVEDD


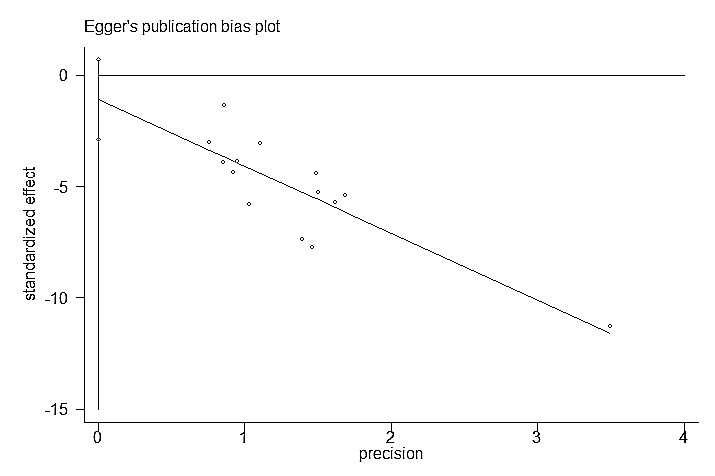


**D.** The Egger’s test of LVESD


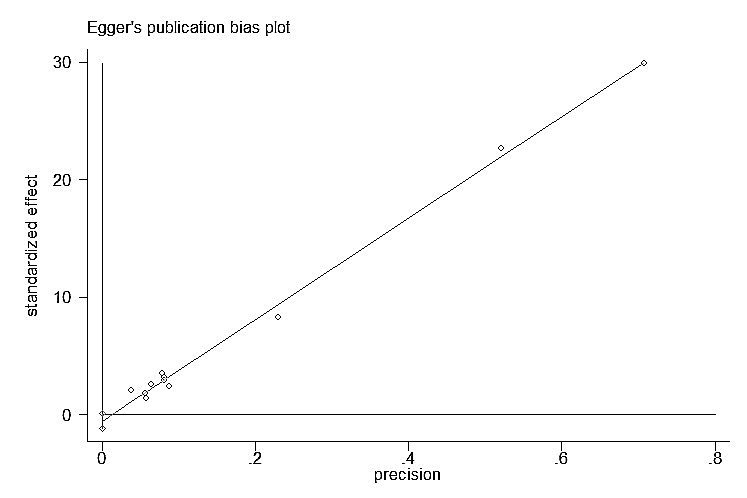


**E.** The Egger’s test of 6MWD


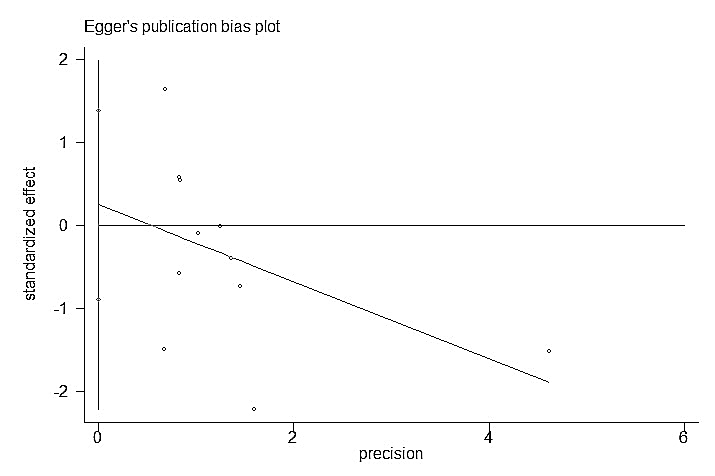


**F.** The Egger’s test of ARs
