# Supplementary material for: Efficacy and Safety of Qili Qiangxin Capsule on Dilated Cardiomyopathy: A Systematic Review and Meta-Analysis of 35 Randomized Controlled Trials
Source: Front Pharmacol. 2022 Apr 28;13:893602. doi: 10.3389/fphar.2022.893602 (PMC9095857; doi:10.3389/fphar.2022.893602)
Supplement: Supplementary file 1 [file DataSheet1.ZIP › table S2.Search Strategies.docx]

**Table S2.** Search Strategies

| **PubMed** | (Qili Qiangxin capsule OR Qili Qiangxin OR qiliqiangxin)  AND  (dilated cardiomyopathy OR dilative cardiomyopathy OR DCM)  AND  (random* controlled trial [pt] OR controlled clinical trial* [pt] OR randomized [tiab] OR placebo [tiab] OR drug therapy [sh] OR random* [tiab] OR trial* [tiab] OR group* [tiab])  NOT  (animals [mh] NOT humans [mh]) |
| --- | --- |
| **Web of Science** | (Qili Qiangxin capsule OR Qili Qiangxin OR qiliqiangxin)  AND  (dilated cardiomyopathy OR dilative cardiomyopathy OR DCM)  AND  (randomized controlled trial [pt] OR controlled clinical trial [pt] OR trial [tiab] OR clinical trials as topic [mesh: noexp] OR Clinical Trial OR random* [tiab] OR random allocation [mh] OR single-blind method [mh] OR double-blind method [mh]) |
| **Clinicaltrials.gov** | Condition or disease: dilated cardiomyopathy OR dilative cardiomyopathy OR DCM  Other terms: (Qili Qiangxin capsule OR Qili Qiangxin OR qiliqiangxin) |
| **the Cochrane library** | 1 Qili Qiangxin capsule  2 Qili Qiangxin '  3 qiliqiangxin  4 1 or 2 or 3  5 dilated cardiomyopathy  6 dilative cardiomyopathy  7 DCM  8 5 or 6 or 7  9 4 and 8  10 randomized controlled trial  11 single blind procedure or double blind procedure  12 10 or 11  13 9 and 12 |
| **CNKI** | ( SU='芪苈强心胶囊')  AND  (SU='扩张型心肌病') |
| **Wanfang** | (芪苈强心胶囊)  AND  (扩张型心肌病) |
| **VIP** | (M=芪苈强心胶囊)  AND  (M=扩张型心肌病) |
| **CBM** | ("芪苈强心胶囊"[常用字段:智能])  AND  ("扩张型心肌病"[常用字段:智能]) |
